# Perspective: Daniela Novick, cytokines and their receptors

**DOI:** 10.3389/fimmu.2023.1160651

**Published:** 2023-05-11

**Authors:** Pietro Ghezzi, Giamila Fantuzzi, Charles A. Dinarello

**Affiliations:** ^1^ Department of Biomolecular Sciences, Università di Urbino, Urbino, Italy; ^2^ Department of Kinesiology and Nutrition, University of Illinois Chicago, Chicago, IL, United States; ^3^ Department of Medicine, University of Colorado Denver, Aurora, CO, United States

**Keywords:** cytokines, cytokine receptors, cytokine antagonists, antibodies, interferon, interleukin - 1

## Abstract

This Perspective highlights the work of Dr. Daniela Novick in the field of cytokine biology. Using affinity chromatography to characterize cytokine-binding proteins, she identified soluble forms of the receptors as well as binding proteins for several cytokines, including tumor necrosis factor, interleukin (IL) 6, IL-18 and IL-32. Importantly, her work has been key in the development of monoclonal antibodies against interferons and cytokines. This Perspective discusses her contribution to the field and highlights her recent review on this topic.

## Introduction

The discovery of cytokines has been a success story in biomedical research. In the late 1970s the interest was in identifying the soluble mediators that hematopoietic cells release to fight cancer or viral infection, or to stimulate other cells *in vitro*. In addition, cytokines were thought to be part of the acute phase response in humans with inflammatory diseases. Multiple biological properties were claimed for the unfractionated supernatants. A conundrum developed with increasing biological activities attributed *in vitro* and *in vivo* assays. Although many reported fractionation methods such as gel filtration, purification was hampered by the unexpected high potency of the soluble mediators. For example, a particular biological activity was observed in gel fractions without visible proteins on standard electrophoresis. With one notable exception, no one purified any of these mediators to homogeneity. In 1981, human growth hormone was produced by *E. coli* using cDNA coding for the growth hormone and ushered in a true milestone in biology. The concept excited many who realized that large amounts of biologically active proteins could be available using the recombinant DNA technology. It was then possible to produce “biologicals”, as they were termed, to fight against cancer. In March 1980, interferon (IFN) made the cover of Time magazine as “the IF drug for cancer”. Interferons produced using recombinant DNA technology were approved for use in the treatment of chronic hepatitis, viral infections and autoimmune disease such as multiple sclerosis.

By the end of 1984, two of the major inflammatory proteins, interleukin 1 (IL-1) and tumor necrosis factor (TNF), were produced by recombinant DNA. Since IL-1 and TNF were too inflammatory for clinical use as anticancer agents, the landscape changed. The focus then shifted to the development of anti-cytokine biologicals (antibodies, soluble receptors and receptor antagonists). This led to the “cytokine theory of disease”, a concept that continues today. Translation into clinical medicine started with commercialization of biologicals by major pharmaceutical companies, for example, the use of the TNF soluble receptor to treat rheumatoid arthritis.

## Daniela Novick’s contribution

The recent review by Dr. Daniela Novick published in Frontiers Drug Discovery (1) highlights her contribution to the cytokine field. Working together with other researchers at the Weizmann Institute, she elegantly developed monoclonal antibodies against IFN-α, -β and -γ and the first anti-IL-6 monoclonal antibody. We should not forget that producing monoclonal antibodies was challenging, pioneering and certainly hardly routine in the early 80’s. It is not an overstatement that the availability of these reagents was highly valuable for the study of cytokines.

The strategy adopted and perfected by Dr. Novick for the isolation of soluble receptors and binding proteins proved a winning formula in yielding the target putative proteins. In her review Dr. Novick highlights the advantages of her strategy and the solutions for the difficulties she confronted throughout her research. She employed ligand affinity chromatography in which the ligand (often a cytokine) is covalently immobilized on Sepharose gel and then a rich mixture of proteins, such as body fluids or cell extracts, is passed over this gel. Soluble receptors and binding proteins have a high affinity for the ligand and therefore the binding is specific. By changing the pH, these proteins dissociate from the gel and are collected. Their identity was determined by N-terminal amino acid sequencing using Edman degradation.

Biologically active cytokines were known to be present in the urine. For example, Jean-Michel Dayer described that urine from patients with fever contain a protein inhibitor of IL-1 (2) and William Arend described a similar inhibitor produced by activated monocytes (3). This urine-derived inhibitor was later identified as the IL-1 Receptor antagonist (4). The recombinant antagonist is now known as anakinra and is used widely to treat IL-1-mediated inflammatory diseases. However, the lesson from Jean-Michel’s laboratory was that inflammatory cytokines have a naturally occurring counterbalance. In the case of TNF, scientists in the laboratory of David Wallach in the Weizmann Institute sought such molecules in concentrated urine that would reduce the activities of the highly inflammatory TNF.

Isolating the putative “TNF inhibitor” from concentrated urine was a major undertaking. However, Dr. Novick offered to help in the search for the then elusive TNF receptor using her method of ligand affinity chromatography. This effort resulted in isolation of a TNF receptor, which established for the first time that there were two TNF receptors (5). At the time, this second receptor was named TNF Binding Protein II (TBPII). As she described in her review (1), a modification of TBPII became a widely used treatment of rheumatoid arthritis and is called etanercept.

In a 1989 article, Dr. Novick and colleagues described isolation of the soluble receptors for IL-6 and IFN-γ from the same concentrated preparation of urine (6). The article contained data demonstrating that the soluble IFN-γ receptor inhibited IFN-γ’s activity, just as it was expected. However, even though it reported isolation of soluble IL-6 receptors, the article made no mention of testing for these molecules’ inhibitory activity. “We actually did that,” Daniela Novick told one of us during an interview. “We obviously expected the soluble receptor to inhibit IL-6 activity just as all the other known soluble receptors did. But it did not. To the contrary, IL-6 soluble receptors potentiated the activity of IL-6. At that time I thought that I did not know how to work! So did Michel Revel, my supervisor. This is the reason why the bioassay is not included in our 1989 publication on soluble cytokine receptors. I still have those lab notebooks.” (Ref. 7, pages 165-166). Later on, in a tour de force, Dr. Novick isolated the soluble form of the ligand binding chain of Type I interferon receptor and its cell surface counterpart (8), later named IFNAR2. She brought to an end over 30 years of search for this receptor. Her findings significantly advanced our understanding the IFN signal transduction and of the role of IFN in disease.

Dr. Novick’s idea that human urine could represent a “golden mine” (her words in a personal communication) for soluble receptors led to the identification of additional soluble receptors and binding proteins. Thus, others sought her expertise. As the biology of new cytokines were reported, their receptors were also sought. One particular case was the cytokine IL-18, formerly called interferon gamma inducing factor. Because of the importance of IFN-γ in innate and adaptive immunity, a considerable investigation involved the mechanism by which IL-18 induced IFN-γ and a search for its receptor. The availability of milligram amounts of recombinant IL-18 enabled Dr. Novick to employ ligand affinity chromatography and to isolate what was then thought to be the IL-18 receptor. However, at the same time, a full-length IL-18 receptor was reported and the sequence did not match the sequence of the urinary isolate. Many attempts of Dr. Novick and her student Soo-Hyun Kim to identify a cDNA encoding a transmembrane version of the protein failed and they concluded that the molecule was exactly that, a binding protein. So they named the molecule the IL-18 Binding Protein (IL-18BP) (9). In their publication, they reported that IL-18BP inhibited the induction of IFN-γ. The affinity of the IL-18BP for IL-18 was unusually high (0.4 nM) and in many ways validated the efficiency of ligand affinity chromatography. In her review (1), Dr. Novick tells of treating children who are born with life-threatening mutations that result in overexpression of IL-18 and how continuous treatment with a recombinant form of IL-18BP, generically termed tadekinig alfa, has enabled them to lead almost normal life.

In her review (1), we learn of the approaches developed by Dr. Novick that led to the identification of several soluble receptors and binding proteins, not only those specific for cytokines (TNF, IL-6, IFNs, IL-18 and IL-32) but also for other key molecules such as LDL and heparanase. Her findings were not only important to characterize the biology of cytokines, but some have also been approved for the therapy of chronic inflammatory diseases – making this story an example of translational medicine.

## Conclusions

By reading the historical accounts of her studies (1), we learn about the strategies Dr Novick used. History aside, we also appreciate how, despite the difficulty of being a woman scientist in a field mostly dominated by men, she contributed so much to blockbuster biological drugs such as Rebif (IFN-β) for multiple sclerosis and etanercept for rheumatoid arthritis, plaque psoriasis, psoriatic arthritis, polyarticular juvenile arthritis and ankylosing spondylitis. She was instrumental in the generation of the first monoclonals to TNF that became the treatment for inflammatory bowel disease. We also learn of the life-saving use of tadekinig alfa. It’s an incredible story that includes also a view on the relevant recent progress and that is published in Frontiers (1).

## Author contributions

PG, GF, CD: Wrote the manuscript. All authors contributed to the article and approved the submitted version.

## References

[1] Novick D. Nine receptors and binding proteins, four drugs, and one woman: historical and personal perspectives. Front Drug Discov (2022) 2. doi: 10.3389/fddsv.2022.1001487

[2] Dayer JM. From supernatants to cytokines: a personal view on the early history of IL-1, IL-1Ra, TNF and its inhibitor in rheumatology. Arthritis Res Ther (2018) 20:101. doi: 10.1186/s13075-018-1607-y 29848388 PMC5977557

[3] Arend WP Joslin FG Massoni RJ. Effects of immune complexes on production by human monocytes of interleukin 1 or an interleukin 1 inhibitor. J Immunol (1985) 134(6):3868–75. doi: 10.4049/jimmunol.134.6.3868 2985700

[4] Arend WP Joslin FG Thompson RC Hannum CH. An IL-1 inhibitor from human monocytes. production and characterization of biologic properties. J Immunol (1989) 143(6):1851–8. 2528582

[5] Engelmann H Novick D Wallach D. Two tumor necrosis factor- binding proteins purified from human urine. Evidence for immunological cross- reactivity with cell surface tumor necrosis factor receptors. J Biol Chem (1990) 265(3):1531–6. doi: 10.1016/s0021-9258(19)40049-5 2153136

[6] Novick D Engelmann H Wallach D Rubinstein M. Soluble cytokine receptors are present in normal human urine. J Exp Med (1989) 170(4):1409–14. doi: 10.1084/jem.170.4.1409 PMC2189483 2529343

[7] Fantuzzi G. Body messages: the quest for the proteins of cellular communication. Cambridge, USA: Harvard University Press (2016).

[8] Novick D Cohen B Rubinstein M. The human interferon alpha/ beta receptor: characterization and molecular cloning. Cell (1994) 77(3):391–400. doi: 10.1016/0092-8674(94)90154-6 8181059

[9] Novick D Kim SH Fantuzzi G Reznikov LL Dinarello CA Rubinstein M. Interleukin-18 binding protein: a novel modulator of the Th1 cytokine response. Immunity (1999) 10(1):127–36. doi: 10.1016/s1074-7613(00)80013-8 10023777

